# Development of a High-Throughput *ex-Vivo* Burn Wound Model Using Porcine Skin, and Its Application to Evaluate New Approaches to Control Wound Infection

**DOI:** 10.3389/fcimb.2018.00196

**Published:** 2018-06-15

**Authors:** Diana R. Alves, Simon P. Booth, Paola Scavone, Pascale Schellenberger, Jonathan Salvage, Cinzia Dedi, Naing-Tun Thet, A. Toby A. Jenkins, Ryan Waters, Keng W. Ng, Andrew D. J. Overall, Anthony D. Metcalfe, Jonathan Nzakizwanayo, Brian V. Jones

**Affiliations:** ^1^School of Pharmacy and Biomolecular Sciences, University of Brighton, Brighton, United Kingdom; ^2^The Blond McIndoe Research Foundation, Queen Victoria Hospital, East Grinstead, United Kingdom; ^3^The Queen Victoria Hospital NHS Foundation Trust, East Grinstead, United Kingdom; ^4^Department of Microbiology, Instituto de Investigaciones Biológicas Clemente Estable, Montevideo, Uruguay; ^5^Electron Microscopy Imaging Centre, School of Life Sciences, University of Sussex, Brighton, United Kingdom; ^6^Department of Chemistry, University of Bath, Bath, United Kingdom; ^7^The Pirbright Institute, Woking, United Kingdom; ^8^School of Pharmacy, Newcastle University, Newcastle Upon Tyne, United Kingdom; ^9^School of Chemical Engineering, University of Birmingham, Birmingham, United Kingdom; ^10^Department of Biology and Biological Sciences, University of Bath, Bath, United Kingdom

**Keywords:** *ex-vivo* burn wound model, infection, *Staphylococcus aureus*, bacteriophage therapy, biofilm, accessory gene regulator, infection responsive materials

## Abstract

Biofilm formation in wounds is considered a major barrier to successful treatment, and has been associated with the transition of wounds to a chronic non-healing state. Here, we present a novel laboratory model of wound biofilm formation using *ex-vivo* porcine skin and a custom burn wound array device. The model supports high-throughput studies of biofilm formation and is compatible with a range of established methods for monitoring bacterial growth, biofilm formation, and gene expression. We demonstrate the use of this model by evaluating the potential for bacteriophage to control biofilm formation by *Staphylococcus aureus*, and for population density dependant expression of *S. aureus* virulence factors (regulated by the Accessory Gene Regulator, *agr*) to signal clinically relevant wound infection. Enumeration of colony forming units and metabolic activity using the XTT assay, confirmed growth of bacteria in wounds and showed a significant reduction in viable cells after phage treatment. Confocal laser scanning microscopy confirmed the growth of biofilms in wounds, and showed phage treatment could significantly reduce the formation of these communities. Evaluation of *agr* activity by qRT-PCR showed an increase in activity during growth in wound models for most strains. Activation of a prototype infection-responsive dressing designed to provide a visual signal of wound infection, was related to increased *agr* activity. In all assays, excellent reproducibility was observed between replicates using this model.

## Introduction

It has been estimated that 175,000 children and adults visit A&E departments for treatment related to burn injuries each year in the UK (National Burn Care Review Committee Report, 2001). Most wounds heal fully with minimal intervention, but 10–20% of those will become infected posing a serious risk to patient health (Wibbenmeyer et al., 2006). Infection delays healing and promotes scarring, increases the use of antibiotics, and greatly increases the cost of treatment (Bowler et al., 2001; Mayhall, 2003; Church et al., 2006; Wounds UK Best Practice Statement, 2013). Colonization of the wound by bacteria begins almost immediately after injury, and usually includes opportunistic pathogenic bacteria from the surrounding environment or the patient's own microbiome (Bowler et al., 2001; Mayhall, 2003; Church et al., 2006; Rafla and Tredget, 2011; Lebeaux et al., 2013). In many cases only a low-level population of microbes will develop and persist in the wound, which poses no significant barrier to healing. However, in some cases the microbial population will expand to levels that overwhelm immune clearance, impede the normal healing process, and lead to invasive infection that requires treatment (Church et al., 2006; Wounds UK Best Practice Statement, 2013).

This clinically relevant infection also often involves the formation of biofilms in the wound bed which present particular challenges to wound management and successful treatment. Biofilms are dense bacterial communities embedded in a matrix of exopolymeric substances which exhibit distinct physiology, phenotypes, and altered gene expression compared to planktonic cells (Donlan and Costerton, 2002; Zhao et al., 2013; Ganesh et al., 2015; Percival et al., 2015). These features of biofilms make them resilient to environmental factors and immune clearance, and also considerably reduces susceptibility to antibiotics and other topical antimicrobial agents (Donlan and Costerton, 2002; Zhao et al., 2013; Ganesh et al., 2015; Percival et al., 2015). Antibiotics typically perform poorly against bacterial biofilms even when constituent cells are fully susceptible to clinically relevant concentrations in a planktonic state. Congruent with these properties of biofilms is the association between biofilm formation in the wound and treatment failure, poor clinical outcomes, and development of chronic non-healing wounds (Zhao et al., 2013; Ganesh et al., 2015; Percival et al., 2015).

The study of biofilm formation in burn wounds and the evaluation of methods to control these infections is often limited by access to relevant and appropriate models. In particular, early stage laboratory studies aimed at screening and developing new antimicrobial or anti-biofilm agents, or providing fundamental molecular genetic insights into bacterial biofilm formation in wounds, are often hampered by a lack of models which afford both reasonable throughput yet provide a good representation of biofilm formation in burn wounds. Ideally, such models should also be low-cost and simple to operate, support high throughput screening, offer good reproducibility, and be compatible with a wide range of established techniques for measuring or visualizing various aspects of microbial physiology, growth, biofilm formation, and gene expression.

Although a number of excellent *in-vivo* models of wound infection have been described and have considerably enhanced understanding of wound biofilm formation, their use is subject to ethical and economic considerations, and relatively few are focused on burn wounds specifically (Dai et al., 2011; Lebeaux et al., 2013; Roy et al., 2014; Ganesh et al., 2015; Seaton et al., 2015). Porcine models are considered to provide the best representation of human skin and wound healing as well as human burn wound infection (Dai et al., 2011; Ganesh et al., 2015; Seaton et al., 2015). However, the use of large animals in research and the impact of burn wound injuries on animal welfare amplify regulatory issues and the costs of studies using these models, restricting access and the types of study in which their use is feasible and justified (Dai et al., 2011; Seaton et al., 2015). Murine models are often more affordable but have similar ethical issues, and are considered to be less representative of human wound infection. The small size of these animals also limits the number of wounds that can be made and statistical robustness that can be achieved (Dai et al., 2011; Ganesh et al., 2015; Seaton et al., 2015).

Conversely, while many *in-vitro* models are simple, affordable, and scalable for screening type applications, and allow study of the main features of biofilms, these often provide a poor representation of the wound environment and are largely restricted to growth of biofilms on abiotic surfaces (Lebeaux et al., 2013; Ganesh et al., 2015). Mammalian cell culture based models which seek to provide biotic surfaces for biofilm formation in the form of cell monolayers are also available, but do not provide good representation of skin structure, are often more costly and difficult to operate, and less well-suited to support large scale screening studies (Lebeaux et al., 2013; Ganesh et al., 2015).

As such, there remains scope to develop additional *in-vitro* or *ex-vivo* models that can support investigation of burn wound infection and biofilm formation at early stages, particularly for studies with a requirement to screen numerous compounds, bacterial isolates, or understand fundamental aspects of biofilm formation. Previously we have described the use of *ex-vivo* porcine skin sections with thermal damage comparable to partial thickness burns, for the study of bacterial biofilm formation and novel infection responsive dressing materials (Thet et al., 2015). This model provides many of the desirable features noted above, and here we extend this work to develop a high-throughput and reproducible *ex-vivo* porcine skin model of wound infection and biofilm formation. We demonstrate the use of this model by evaluating the potential for bacteriophage therapy to control biofilm formation in this setting, and the potential to exploit population density dependent changes in virulence gene expression for early detection of clinically relevant infection.

## Materials and methods

### Bacteria and growth conditions

Bacteria were cultured in Tryptone Soy Broth (TSB) at 37°C with shaking, or on TSB solidified by addition of 15 g/L Technical agar (TSA). Soft agar overlays, used for phage enrichments, purification, and enumeration, were derived from TSB and contained 6.5 g/L Technical agar (S-TSA). All chemicals, reagents and growth media were obtained from Fisher Scientific (UK), or Sigma (UK) unless otherwise stated. *Staphylococcus aureus* strains used in this study are described in Table 1, and represent a range of hospital or community acquired methicillin-resistant *S. aureus* (MRSA), epidemic MRSA (EMRSA), and methicillin-sensitive *S. aureus* (MSSA) from active infection. Spontaneous rifampicin-resistant mutants of MRSA252 were generated by serial passage on TSB agar supplemented with increasing concentrations of rifampicin from 5 to 100 μg/mL. All work involving bacterial pathogens was conducted in containment level 2 laboratories and in compliance with institutional guidelines and UK legislation on Containment of Substances Hazardous to Health.

**Table 1:** Bacterial strains and bacteriophage used in this study.

***S. aureus* Strain/Phage**	**Description**	**Reference/Source**
RN6390B	NTCT8325 cured of three prophages. Agr^+^ wild type strain capable of lysing lipid vesicles	Peng et al., 1988; Laabei et al., 2014
RN6911	Isogenic agr knockout mutant of RN6390B, incapable of lysing lipid vesicles	Sakoulas et al., 2009; Laabei et al., 2014
B4328	Clinical wound isolate. Use to isolate and propagate phage SAB4328-30	This study—Royal Sussex County Hospital
MSSA 3 MSSA 10 MSSA 16 MSSA 21 MSSA 49 MSSA 52 MSSA 56 MSSA 67 MSSA 69 MSSA 71 MSSA 114 MSSA 160 MRSA 82 MSSA 476 MSSA 707 MSSA 15981 MRSA 252 MRSA 378 MRSA 455 EMRSA 1 EMRSA 9 EMRSA 13 EMRSA 15 EMRSA 16	Strains from patients with invasive *S. aureus* infection collected between 1997 and 1998 in Oxford UK (both hospital and community acquired isolates)	Enright et al., 2000
USA300	Community acquired epidemic clone of methicillin resistant *S. aureus*	McDougal et al., 2003
15981	Clinical *S aureus* isolate used to propagate phage DRA88	Alves et al., 2014
MRSA252-Rif^R^	Derivative of MRSA252 rendered resistant to rifampicin through serial sub-culture	This study
Phage DRA88	*S. aureus* phage capable of infecting a broad range of clinical isolates	Alves et al., 2014
Phage SAB4328-A	Isolated against *S. aureus* B4328	This study

### Phage isolation and purification

Phage DRA88 has previously been isolated by the authors (Alves et al., 2014). Phage SAB4328-A was isolated from sewage collected from wastewater treatment plants in the UK (Hailsham North WWTP, East Sussex), by performing bacterial enrichments with a recent *S. aureus* clinical isolate (from Royal Sussex County Hospital, UK). Hundred milliliters of wastewater was mixed with 387.5 mL of LB, and inoculated with 2.5 mL of an overnight culture of *S. aureus* B4328. This was incubated at 37°C overnight without shaking. Ten milliliters of aliquots were centrifuged (3,000 × g for 30 min), and supernatants filtered into fresh sterile tubes using 0.45 μm pore syringe filters (Sartorius, UK). Hundred microliters of supernatant (containing enriched phage) were mixed with 100 μL of host culture and incubated for 5 min at room temperature, then mixed with 3 mL S-TSA. The S-TSA mixture was poured over TSA plates to create overlays, and incubated at 37°C overnight once set. Single well-isolated plaques with different morphologies were picked off and resuspended in 300 μL SM Buffer (100 mM NaCl, 10 mM MgSO_4_.7H_2_O, 50 mM Tris-HCl pH 7.5, 0.01% gelatine), the phage suspension serially diluted, and plated on S-TSA plates containing host bacteria as described above. The process was repeated until bacterial lawns showed homogeneity of plaque morphology. Finally, an individual plaque was picked off and resuspended in SM buffer for use in subsequent experiments.

### Preparation of high phage stocks

For the preparation of high titer stocks, phage SAB4328-A was propagated on *S. aureus* B4328, and phage DRA88 was propagated on *S. aureus* 15981. Briefly, 100 μL of phage suspension and 100 μL of host culture were mixed and left for 5 min at room temperature. The bacterial and phage mixture was then added to 3 mL of S-TSA, mixed gently, and poured over TSA plates. After overnight incubation at 37°C, plates displaying confluent lysis were selected and 3 mL of SM buffer supplemented with 2% (v/v) chloroform were added before incubation at 37°C for 4 h. High-titer phage solution was removed from the plates, centrifuged (8,000 × g for 10 min) to remove cell debris, and then filter-sterilized (pore size, 0.22 μm). In order to concentrate the phage lysate 20% PEG-8000 solution supplemented with 1 M NaCl to a final concentration of 10% was added to the lysate and incubate at 4°C overnight. The mixture was centrifuged at 8,000 × g for 20 min, the pellet resuspended in SM buffer and 1/5 volume chloroform added, followed by a slow centrifugation to collect the aqueous phase with phage. The suspension was then stored at 4°C for further use.

### Electron microscopy

Purified phage particles at a concentration of 10^9^ PFU/mL were deposited on 300 mesh carbon-coated copper grids (Agar Scientific, UK) and negatively stained with 1% uranyl acetate (pH 4). The surface of the copper grids were ionized for 2 min immediately prior to sample deposition, 5 μL of phage lysate were spotted on the surface of the grid and allowed to stand for 1 min, followed by 30 s negative staining and air dried. Visualization was performed using a transmission electron microscope JEOL JEM-1400Plus, operated at 120 kV (pixel size = 0.1 nm), equipped with a Gatan OneView 4K CMOS digital camera. For measurement estimations 10 images of each phage were taken.

### *Ex-vivo* burn wound model set up

#### Burn wound array device (BWAD)

**Figure 1:**
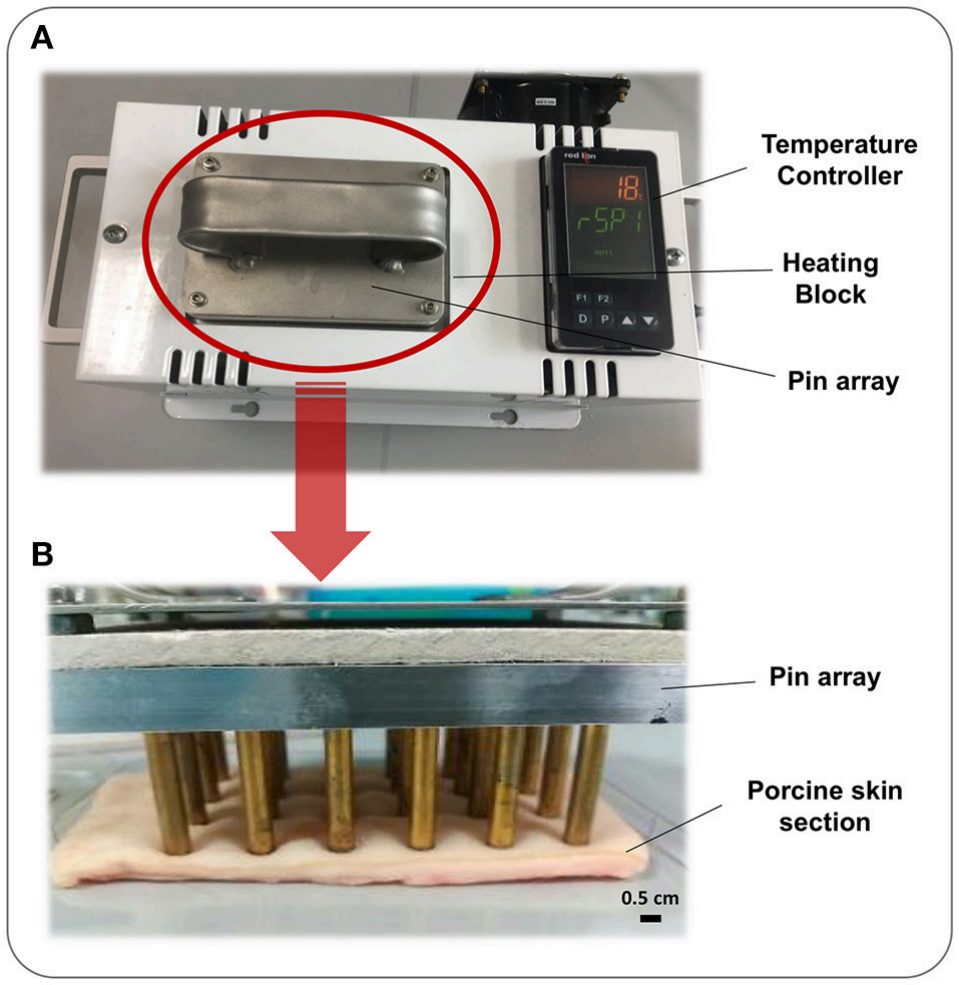
Burn Wound Array Device. The Burn Wound Array Device consists of an array of 24 brass pins that rests in a temperature controlled heated block. Pins are heated to the set temperature and the device signals the user when this has been reached and tool is ready for use. **(A)** Heated block and temperature controller with 24 pin array inserted and heating. **(B)** 24 pin array resting on a section of porcine skin to generate thermal injuries.

The device consists of 24 brass pins of 5 mm diameter housed in a single weighted block. This array is inserted in a heated block, which elevates the temperature of each pin to the desired temperature as specified by the user (Figure 1). The pin array may then be used to generate a grid of 24 burn wounds derived from exposure of skin to pins with identical temperature, time, and pressure.

#### Burn model preparation

**Figure 2:**
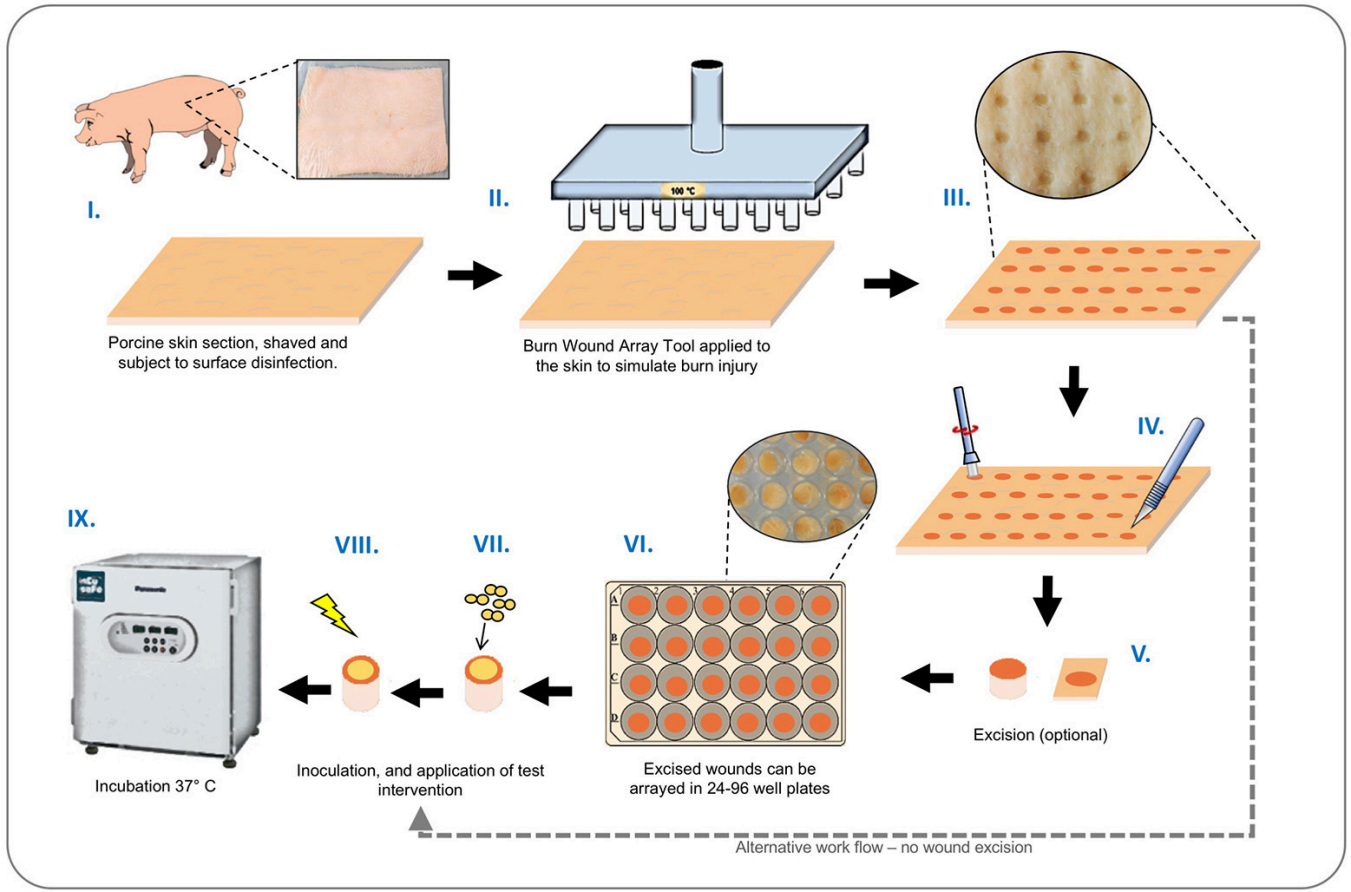
*Ex-vivo* burn wound model workflow. Graphical representation showing main stages of set-up and operation of the *ex-vivo* porcine burn wound model used in this study. **(I)** Sections of porcine skin are initially prepared by shaving and surface disinfection in 70% ethanol solution; **(II, III)** The BWAD (see Figure 1) is used to generate an array of consistent partial thickness burn injuries on porcine skin; **(IV)** If isolation of wounds is required, individual wounds may be excised using a punch biopsy and transferred into wells of tissue culture plates. Wound arrays may be used directly on skin sections without excision. **(V–VIII)** Individual wounds are inoculated with test organisms to simulate infection and wound biofilm formation. Potential treatments may be evaluated against a subset of infected wounds in the array. **(IX)** Infected wounds are incubated under required conditions and biofilm formation, bacterial growth, and impact on interventions tested can be evaluated as appropriate. Subsequent methods demonstrated in this study are: Recovery and enumeration of viable cells, quantification of metabolic activity via XXT reduction, the selective staining of biofilms followed by direct imaging of biofilms through CLSM and measurement of fluorescence intensity, extraction of RNA, and quantification of gene expression by qRT-PCR.

A schematic illustrating the basic set-up and operation of our *ex-vivo* model is provided in Figure 2. Porcine skin used for this study was supplied by Pirbright Institute (Surrey, UK) as surplus/waste tissue from other experiments, or purchased from Fresh Tissue Supplies Ltd. (Etchingham, UK). Tissues from Pirbright were derived from Large White or Landrace cross hybrid pigs (~10 kg and of both sexes) from a commercial farm. The use of porcine skin in this study was compliant with relevant UK and EU legislation, and approved by the Pirbright Animal Welfare and Ethical Review Board. Skin sections were prepared by shaving, and rinsing under sterile deionized water. The skin was sectioned into 10 cm^2^ segments and the surface disinfected with a solution of 70% ethanol, and allowed to dry in a laminar flow cabinet. The BWAD was pre-heated to 100°C and when at temperature the pin array was placed in contact with the skin for 60 s to create a grid of burn wounds. To avoid cross contamination of wounds during biofilm growth and treatment evaluation, individual burn wounds with surrounding undamaged tissue were excised from the skin section and transferred to individual wells of 96 or 24 well plates as required.

#### Evaluation of bacterial biofilm formation and impact of phage therapy

To evaluate the growth of *S. aureus* biofilms in the *ex-vivo* model we used *S. aureus* strain MRSA252 rendered resistant to rifampicin (MRSA252-Rif^R^), in order to facilitate specific selection and enumeration of this strain at experimental end points. Wounds prepared as described in model operation (Figure 2) were excised using a sterile 6 mm punch biopsy and treated with 100 μg/mL rifampicin to suppress commensal flora. Excess liquid was removed and wounds inoculated with 5 μL of *S. aureus* culture containing 10^4^ CFU. Plates containing explants were sealed in humidified bags and incubated at 37°C for the duration of experiments. To determine the potential for phage to control *S. aureus* biofilm formation in this model, inoculated wounds were treated with doses of phage at set intervals, and bacterial growth and biofilm formation compared with untreated controls at the end of experiments. Two distinct treatment regimes were evaluated: (i) A single phage treatment 4 h after inoculation of *S. aureus* in wounds, and evaluation of biofilms at 24 h; (ii) 2 phage treatments at 4 h, and 24 h post inoculation of *S. aureus* in wounds, and evaluation of biofilms at 48 h. Phage treatments were introduced to wounds as 5 μL volumes containing 10^6^ pfu/mL at 4 h treatment, and 10^7^ pfu/mL at 24 h treatment. Negative controls consisted of both infected and uninfected wounds treated with PBS only.

#### Evaluation of agr activity and activation of infection-responsive dressings

**Figure 3:**
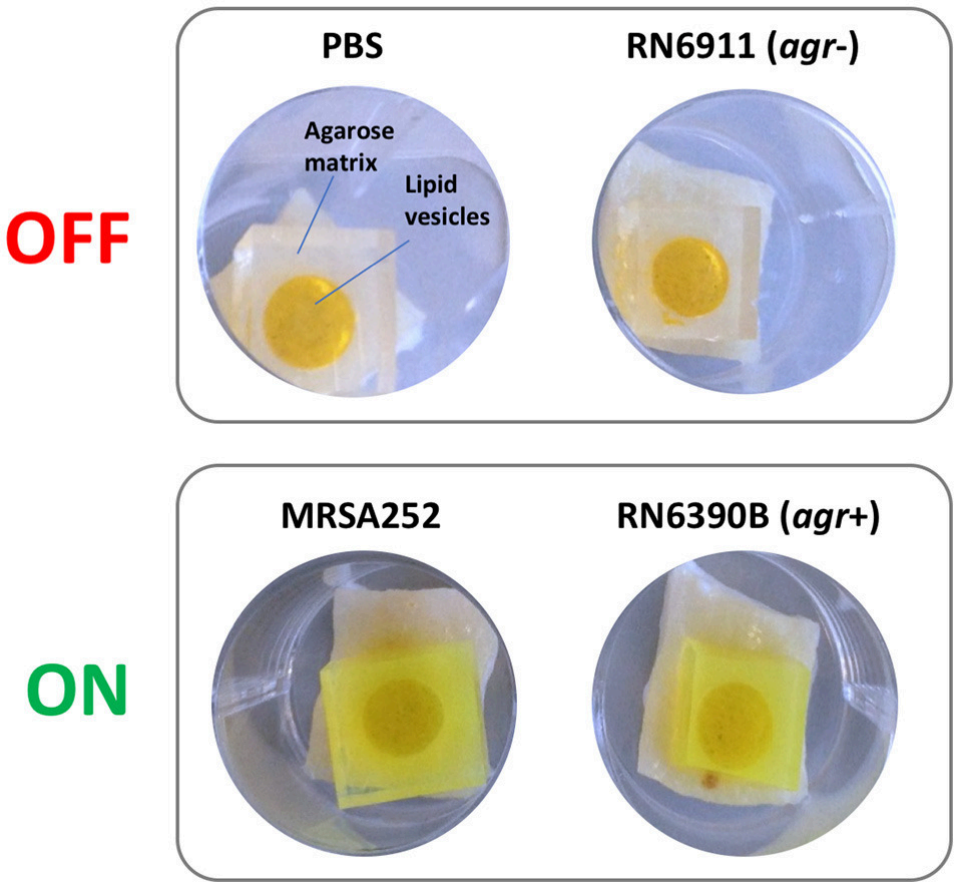
Activation of infection responsive dressings. Examples of non-activated (OFF) and activated (ON) prototype infection responsive dressings originally described by Thet et al. (2015). Dressings consist of carboxyfluorescein loaded lipid vesicles embedded in an agarose matrix. At high concentrations in vesicles, carboxyfluorescein exhibits self-quenching properties that suppresses visible color. Lysis of vesicles by bacteria liberates the dye which diffuses through the dressing leading to development of a bright green color visible to the naked eye. In examples above, dressings incubated for 6 h in wound models with PBS or *S. aureus* RN6911 (which is deficient in the Accessory Gene Regulator agr), show no dressing activation. In contrast, dressings incubated with MRSA252 and RN6390B (parental strain of RN6911 with functional agr) show clear dressing activation.

Correlations between activation of prototype infection responsive dressings (described by Thet et al., 2015) and the activity of the population density controlled virulence gene regulator *agr*, were determined for 28 strains of *S. aureus* (Table 1). For this assay, the porcine skin was prepared as described above, and wounds were generated as described above but excised as 0.5 cm^2^ sections with a scalpel, transferred to 24 well plates, and inoculated with 10^8^ CFU of *S. aureus* in a 20 μL volume, from fresh semi-log phase cultures at OD_600_ of 0.5. Wounds were subsequently overlayed with sections of prototype dressing consisting of carboxyfluorescein loaded lipid vesicles embedded in a hydrogel matrix (Thet et al., 2015). Plates were sealed in humidified plastic bags and incubated for 6 h at 37°C, 5% CO_2_. Dressing activation was evaluated subjectively by eye based on dressing color change as either activated (“on”) or as having no discernible color change (“off”). Figure 3 shows examples of activated (“on”) and non-activated (“off”) dressings. *Agr* activity in corresponding wounds was evaluated by quantitative reverse transcriptase real time PCR (qRT-PCR) as described below.

### Histology of porcine skin

To confirm that damage consistent with a burn injury was generated by the BWAD, skin subjected to thermal injury was prepared for tissue sectioning and compared with uninjured skin from the same section. Excised wounds or regions of uninjured skin were immersed in Optimal Cutting Temperature compound, frozen in liquid nitrogen and 8 μm thick sections removed using a cryostat. Sections were placed onto Vectabond treated glass slides and stored overnight at room temperature. Sections were subsequently immersed in hematoxylin solution for 10 min, followed by removal of excess stain by rinsing in deionized water, and application of 0.1% acid alcohol for 1 s. Slides were again washed with deionized water before immersion in 1% eosin for 5 min. Slides were subjected to a final wash with water before dehydration, mounting in DPX, and microscopic examination.

### ESEM imaging of skin sections

Excised wounds or undamaged skin were directly viewed under the microscope without any fixation or staining. Images were acquired using a Zeiss-Evo LS15 environmental scanning electron microscope (ESEM) (Carl Zeiss Ltd.) operating in extended pressure (EP) mode, and using the following parameters and conditions: 100 μm upper EP aperture, 500 μm lower EDS EP aperture, a chamber pressure of 560–600 pa with c. 85% humidity, an accelerating voltage of 20 kV EHT and using a five quadrant backscatter detector.

### Enumeration of bacteria and phage in wound models

To enumerate bacterial cells and phage particles in wound models, skin explants were immersed in centrifuge tubes containing 1 mL sterilized PBS supplemented with 0.05% Tween-80 and sonicated for 5 min at 60 Hz in an ultrasonic bath to ensure complete dispersal of cells. Subsequently, tubes were vortexed for 10 s before skin explants were removed, and cells pelleted by centrifugation for 5 min at 8,000 × g. For enumeration of phage particles, the resulting supernatant was removed and subject to serial dilution and used to enumerate phage titers using agar overlays seeded with host strains (as described for phage isolation). For bacterial enumeration, the bacterial pellet was resuspended in 1 mL PBS and serial dilutions performed before plating on agar plates supplemented with rifampicin (100 μg/mL).

### XTT assay

Viable bacterial cells in wound biofilms were measured using an XTT [2,3-Bis-(2-Methoxy-4-Nitro-5-Sulfophenyl)-2*H*-Tetrazolium-5-Carboxanilid] assay. The surfaces of excised skin sections were washed twice with PBS to remove non-adherent bacterial cells and placed in a 96-well microtiter plate. Freshly prepared solutions of 1 mg/mL XTT in sterile PBS and 0.4 mM menadione in acetone were combined at a ratio of 20:1 (v/v) XTT:menadione, and protected from light until required. Explants were then immersed in 158 μL PBS and 42 μL XTT:menadione solution, plates wrapped in foil to protect from light exposure, and incubated for 3 h at 37°C. Following incubation, explants were removed from wells and discarded, and the colorimetric changes in the remaining solution measured at 490 nm.

### Confocal laser scanning microscopy

Excised wounds were inoculated with *S. aureus* MRSA252-Rif^R^ and incubated for 24 h as described above for evaluation of bacterial biofilm formation and impact of phage therapy. Excised wounds were then washed twice by immersion and gentle shaking in 1 mL PBS to remove non-adherent cells before staining with 2 μg/mL DAPI solution and 5 μg/mL of Wheat Germ Agglutinin conjugated to Alexa Flour 488 (WGA-488; Invitrogen). WGA-488 binds to Poly-*N*-acetylglucosamine residues present in the *S. aureus* biofilm exopolysaccaride matrix which permits more specific quantification of biofilm formation (Burton et al., 2007; Skogman et al., 2012). The surface of the skin explants was immersed in the staining solution and incubated at room temperature for 20 min in the dark. Following staining, skin explants were washed twice in 1 ml PBS and fixed with 4% paraformaldehyde for 30 min at room temperature in the dark. Skin explants were finally rinsed with 1 ml PBS three times. Images were acquired using a Leica TCS SP5 confocal laser scanning microscope system using a 20 × magnification objective. Intensity of fluorescence signals specific to DAPI and WGA-448 were analyzed using Fiji-ImageJ software. Images at 1,024 × 1,024 resolution and signal intensity data were acquired from three randomly selected regions of each skin sample, with three distinct biological replicates analyzed.

### Measurement of virulence gene expression in wound models

Basal toxin gene expression and toxin gene expression in wound infections were evaluated using Quantitative real-time reverse transcription polymerase chain reaction (qRT-PCR) of *agr*RNAIII toxin gene (Laabei et al., 2014) in *S. aureus* PBS-suspension cells (before inoculation), and cells recovered from the 6-h porcine skin wound model, respectively. The PBS-suspended cells were supplemented with 500 μL RNA*later* stabilization solution; dressings were removed from the wells, and 500 μL RNA*later* was added to each well, and well content mixed, transferred to sterile microcentrifuge tubes, mixed by vortex for 3 min and sonicated at 60 Hz for 5 min. The porcine skin tissue was then removed and the RNA*later*-suspended cells were kept at −80°C waiting for RNA extraction.

Total bacterial RNA was extracted and purified using the MoBio PowerMicrobiome™ RNA kit, and the MoBio DNase Max™ kit (Cambio Ltd., Cambridge, UK), respectively, according to manufacturer's instructions. The treated RNA was used to generate cDNA using the Qiagen QuantiTect Reverse Transcription kit (Qiagen, Manchester, UK) according to manufacturer's instructions. The RT-qPCR was performed on the Rotor gene Q thermocycler using the Rotor-Gene SYBR Green PCR Kit (Qiagen) according to manufacturer's instructions. The gene of interest (GOI) *agr*RNAIII was amplified using previously published primers (Ferreira et al., 2013), RNAIII-F: 5′-AATTTGTTCACTGTGTCGATAAT-3′ and RNAIII-R: 5′-TGGAAAATAGTTGATGAGTTGTT-3′. The *S. aureus* housekeeping (HKP) gene DNA gyrase subunit B (*gyrB*) was amplified using primers gyrB-F: 5′-GGTGGCGACTTTGATCTAGC-3′, and gyrB-R:5′-TTATACAACGGTGGCTGTGC-3′ by Labandeira-Rey et al. (2007).

The PCR reactions were performed in a total volume of 25 μL, which contained 1x Rotor-Gene SYBR Green PCR Master Mix, 5 pmol each of forward and reverse primer, and 150 ng cDNA. For no template negative controls (NTC) cDNA was omitted. PCR products containing GOI and HKP genes were cloned into the pGEM-T Easy plasmid vectors, and were subsequently used to create a 5-point 10-fold serial RT-qPCR standard curve in order to establish efficiencies of RT-qPCR reactions. The standards also served as PCR calibration samples. Reactions were carried out using the following conditions: Polymerase activation at 95°C for 5 min, followed by 40 cycles of two-step cycling, denaturation at 95°C for 5 s and a combined annealing and extension at 60°C for 10 s. Fluorescence was acquired at the end of each combined annealing and extension steps. For each sample three independent biological replicates were assessed, each with two technical replicates per PCR run. Efficiency (E) and C_t_ (threshold cycle) values of RT-qPCRs were calculated using Qiagen RotorGene software. Expression of *agr*RNAIII was normalized to the expression levels of *gyrB* using the modified delta-delta Ct method to include the assay efficiency for each gene, as described by Pfaffl (2001), and according to the formula below:

$$\begin{array}{l} \text{Normalized relative quantity}\\ \;=\frac{{\left(E\text{GOI}\right)}^{\text{ΔCt GOI }\left(\text{calibrator}-\text{Sample}\right)}}{{\left(E\text{HKP}\right)}^{\text{ΔCt HKP}\left(\text{calibrator}-\text{Sample}\right)}} \end{array}$$

where GOI, *agr*RNAIII and HKP, *gyrB*.

### Statistical analyses

All statistical analysis was performed using Prism 6.0c For Mac OS X (GraphPad Software Inc., USA). Data was analyzed using either Student's *t*-test, Mann Whitney Test (for non-parametric data), or the Kruskal-Wallis test with Dunn's correction for multiple comparisons with.

## Results

### Confirmation of burn-like thermal injury on *ex-vivo* porcine skin

**Figure 4:**
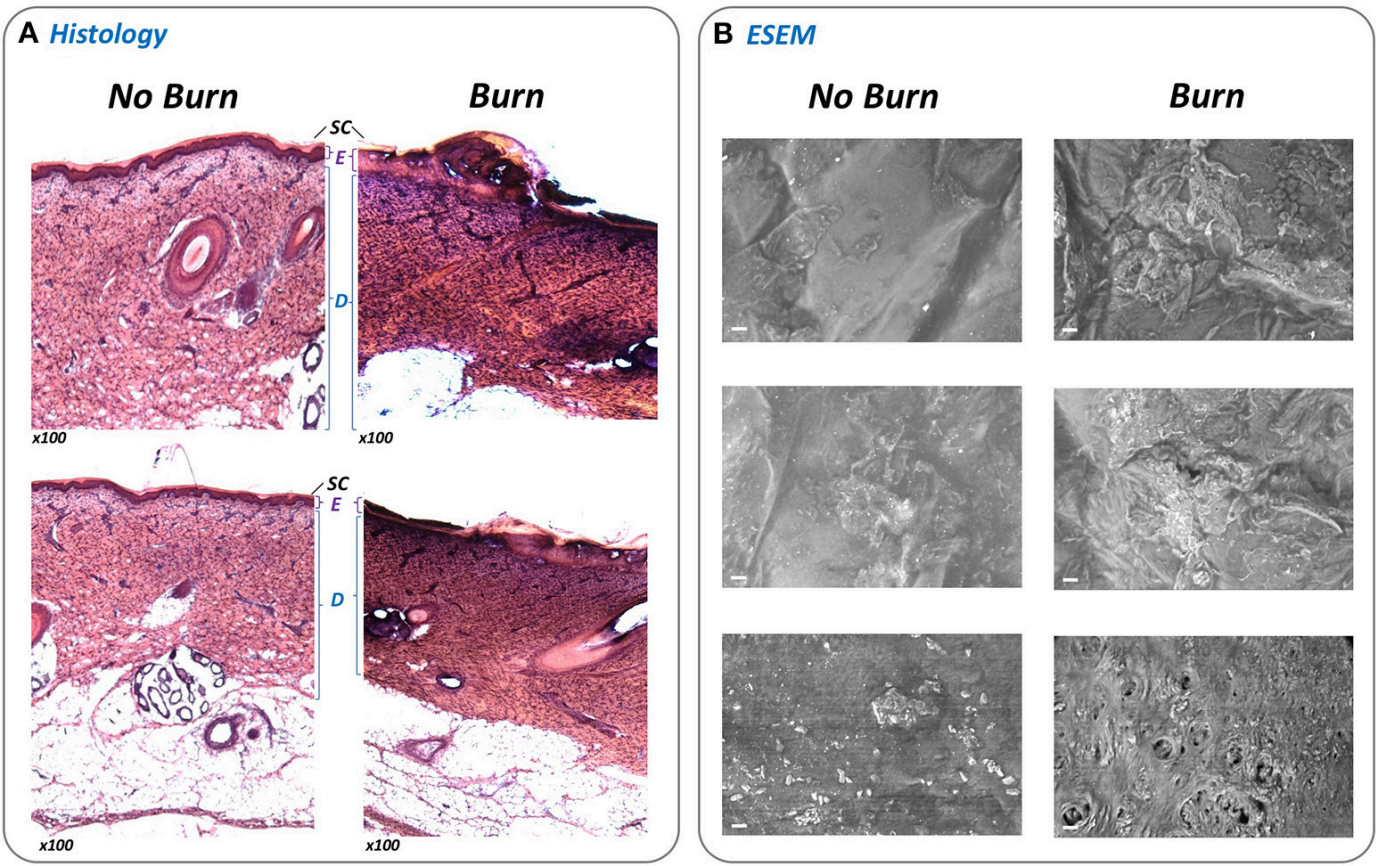
Evaluation of simulated burn wounds. To confirm that the BWAD was able to generate representative thermal injuries in *ex-vivo* porcine skin sections, areas of skin in contact with the BWAD after heating to 100°C were examined by histology and directly by environmental scanning electron microscopy (ESEM), and compared to uninjured skin. **(A)** Thin sections of undamaged and burned skin subjected to H&E staining and histological analysis (x100 magnification). The stratum corneum (*SC*), Epidermis (*E*), and dermis (*D*) are clearly visible and discernible on the undamaged skin sections. In contrast, these structures are disrupted in skin section from BWAD exposed skin, consistent with partial thickness burn injuries. **(B)** ESEM of the surface of undamaged porcine skin and areas in contact with the BWAD. Scale bar = 20 μm.

Central to the standardization and operation of the *ex-vivo* wound model is a bespoke device designed to permit generation of an array of 24 uniform burn wounds on sections of porcine skin (Figures 1, 2). To confirm that the Burn Wound Array Device (BWAD) was able to generate thermal injuries akin to common burn injuries, and in line with our previous low-throughput *ex-vivo* model (Thet et al., 2015), sections of undamaged skin were compared to BWAD treated skin by Environmental Scanning Electron Microscopy and histological analyses (Figure 4). Histopathology showed disruption of the stratum corneum and damage to the underlying epidermis in skin heated with the BWAD, consistent with a partial thickness second degree burn wound (Figure 4A). Areas of skin in contact with the BWAD also showed distinct differences by ESEM, with “burned” regions appearing to exhibit damage and fistulae not observed on skin unexposed to the BWAD (Figure 4B).

### Effects of phage treatment on simulated *S. aureus* wound infection

**Figure 5:**
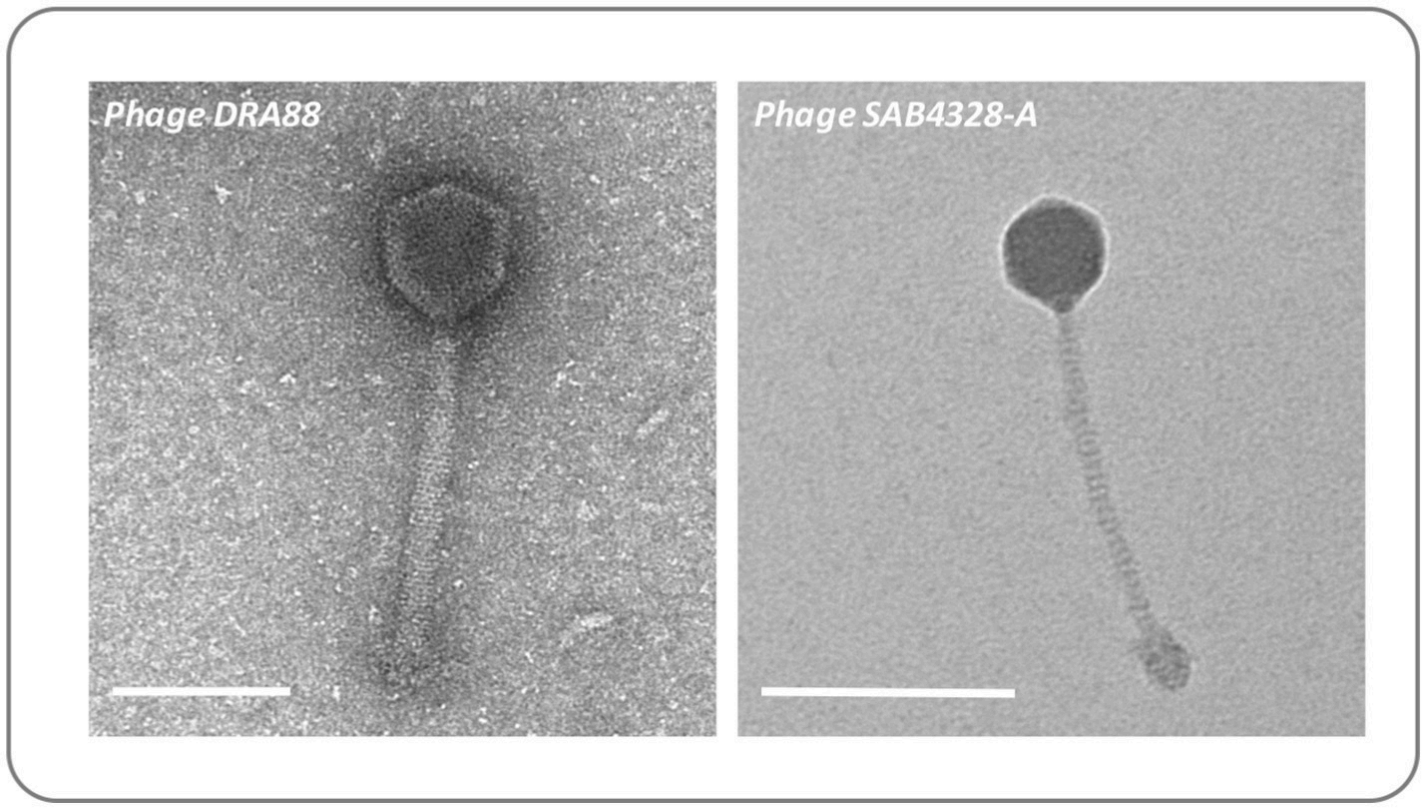
Morphology of bacteriophage used in wound models. The previously isolated phage DRA88 (Alves et al., 2014), and the newly isolated phage SAB4328-A were examined by transmission electron microscopy. Characteristics of the DRA88 virion were congruent with membership of the *Myoviridae* family as previously described, while characteristics of SAB4328-A were in keeping with membership of the *Sipohoviridae* family. Scale bar represents 100 nm.

To demonstrate how the *ex-vivo* model may be used to evaluate interventions for wound infection, we next used this system to explore the capacity for *S. aureus* to grow within wounds created by the BWAD, and the potential for bacteriophage to control these infections. The model was operated as outlined in Figure 2 using an antibiotic resistant clinical isolate of *S. aureus* rendered resistant to rifampicin (referred to as MRSA252-Rif^R^). This allowed us to distinguish inoculated cells from any background skin flora and permitted selective recovery and enumeration. MRSA252-Rif^R^ was confirmed to be susceptible to the previously isolated *S. aureus* phage DRA88 (Alves et al., 2014), and phage SAB4328-A isolated in this study. TEM characterization of these phage were congruent with previous descriptions of DRA88 as a *Myoviridae* (Alves et al., 2014; Figure 5), and showed the newly isolated SAB4328-A to be a member of the *Siphoviridae* family (Figure 5).

**Figure 6:**
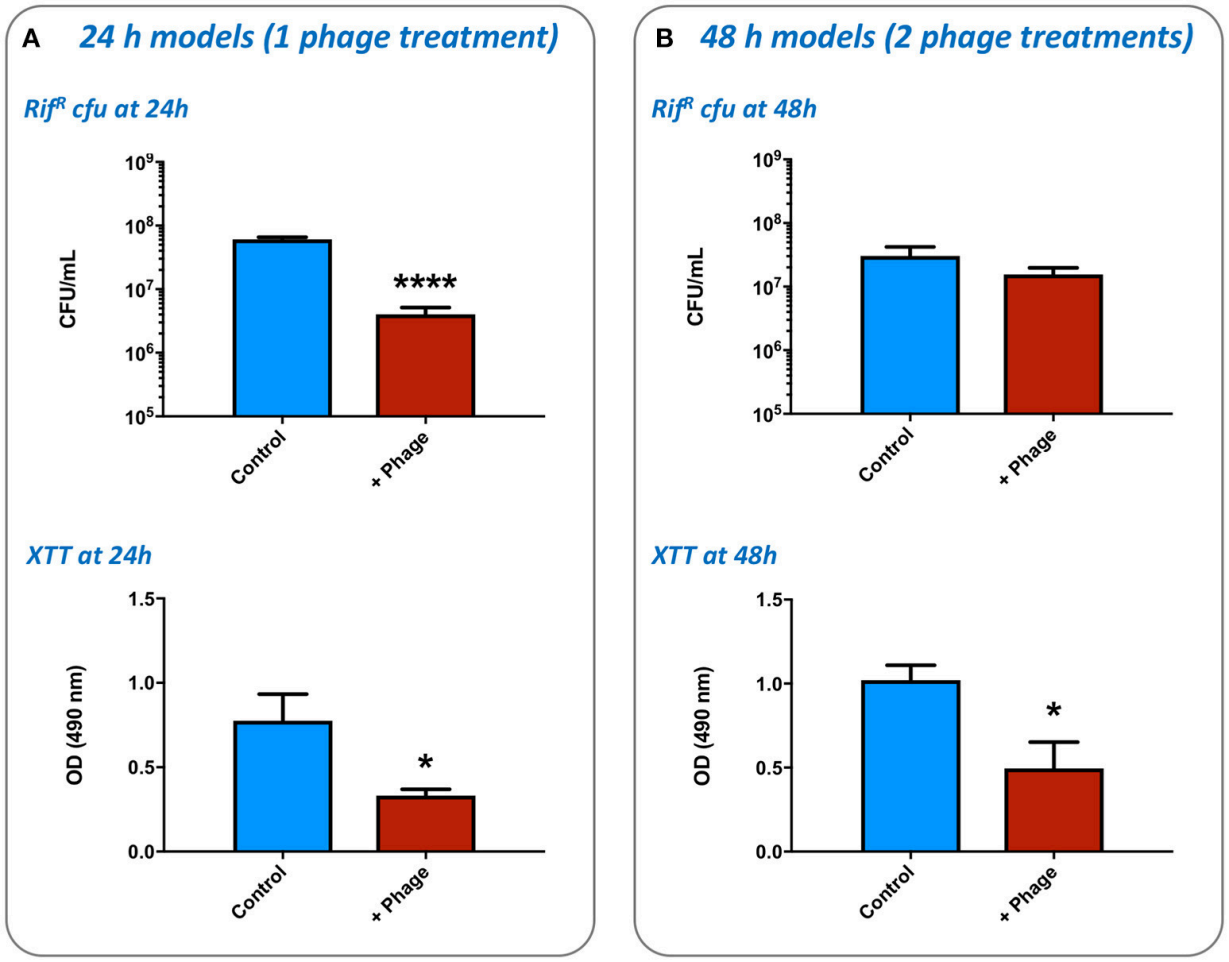
Impact of bacteriophage treatment on simulated wound infection. The potential for bacteriophage to control the growth of bacteria in burn wounds was evaluated using the *ex-vivo* porcine skin model as outlined in Figure 2. The effects of phage treatment were assessed by recovery and enumeration of viable cells, as well as estimates of cellular metabolic activity as measured by the XTT assay, in phage treated vs. untreated wounds inoculated with the multidrug resistant *S. aureus* MRSA252. **(A)** Evaluation of a single phage treatment. Wounds were treated with a single dose of phage 4 h after inoculation and levels of viable bacterial cells assessed after a total of 24 h incubation. **(B)** Evaluation of two phage treatments. Wounds were treated with an initial dose of phage 4 h after inoculation, and again after 24 h of incubation. Levels of viable bacterial cells were assessed after a total of 48 h incubation. Data shows the mean of at least three independent replicates, and error bars show standard error of the mean. Statistically significant differences are denoted by: ^*^P ≤ 0.05, ^****^P < 0.0001.

To determine the growth of *S. aureus* within the wound, and the impact of phage treatment on these simulated wound infections, numbers of viable Rif^R^ cells were enumerated, and levels of bacterial metabolic activity in wounds measured by the XTT assay. The application of a single dose of phage at 4 h post-inoculation (1:1 of each phage) was shown to result in a significant reduction in numbers of Rif^R^ cells and overall metabolic activity compared with untreated controls, after models had run for 24 h (Figure 6A). Enumeration of phage present in these wounds after 24 h also showed higher numbers were present in the wound than originally inoculated (17.25-fold increase) indicating phage replication. In wounds subject to a second phage treatment 24 h after wound inoculation (and run for a total of 48 h), phage treatment did not result in a significant drop in numbers of Rif^R^ cells as measured by viable cell counts (Figure 6B), but overall metabolic activity remained significantly reduced in phage treated wounds (Figure 6B). The number of phage recovered from 48 h models was also considerably higher than the cumulative titer of phage applied to these models at the 4 h and 24 h time points (64.64-fold increase), as well as the titer of phage recovered at the end of 24 h models (41.10-fold higher at 48 h vs. 24 h), indicating phage continued to replicate in models run for longer periods of time.

### Visualization of biofilm formation in wound models and impact of phage treatment

**Figure 7:**
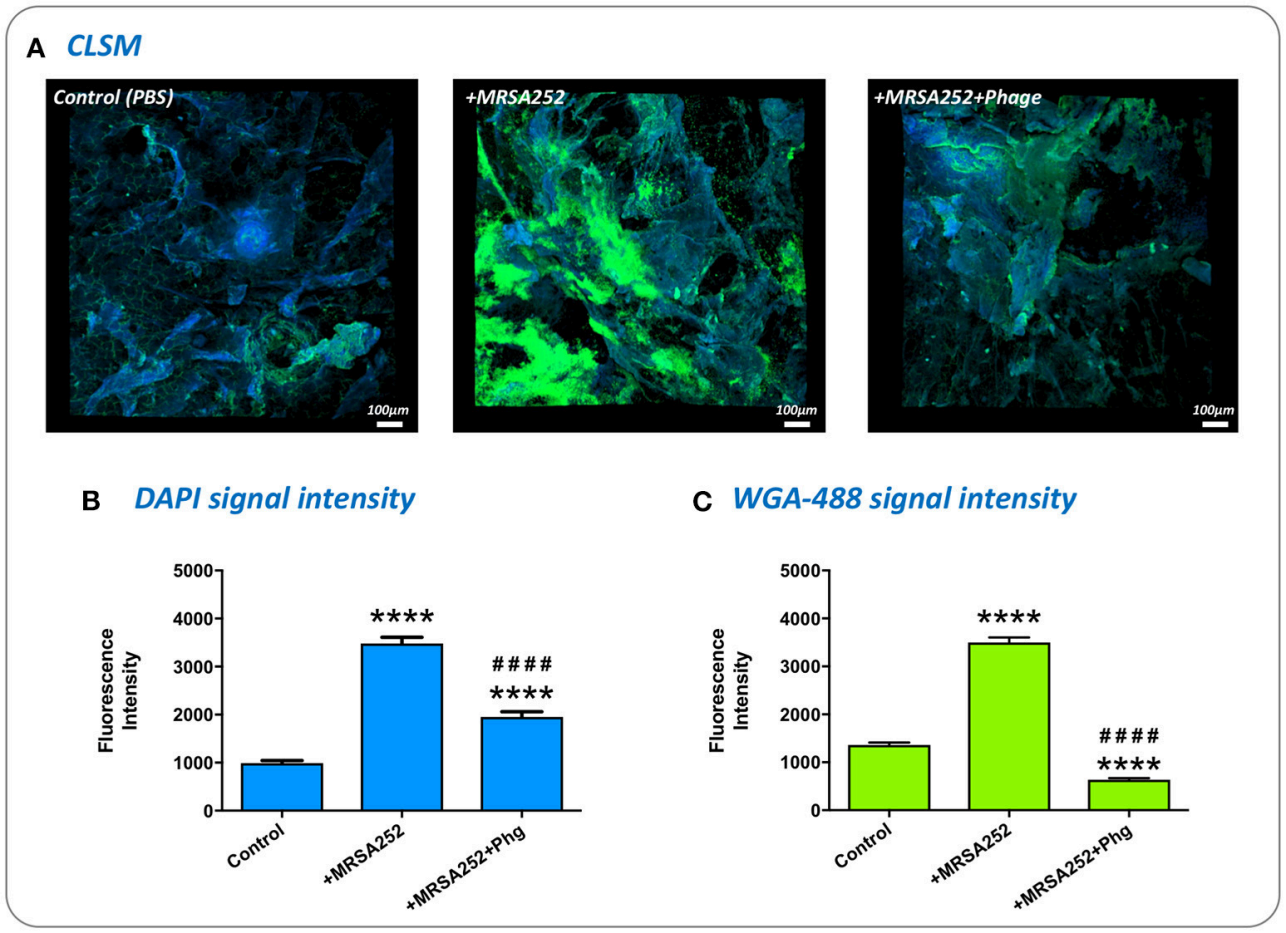
Visualization of biofilm formation in wound models and impact of phage therapy. To confirm biofilm formation in the *ex-vivo* wound model and assess the impact of phage treatment, excised wounds were stained with the general DNA stain DAPI, and WGA-488 fluorescent-lectin conjugate (Wheat Germ Agglutinin with Alexa Flour 488) which binds to poly-*N*-acetlyglucosamine residues in *S. aureus* biofilm matrix. **(A)** Visualization of infected and uninfected wounds by confocal laser scanning microscopy. DAPI staining appears as blue, while WGA-488 staining appears as green areas which are indicative of *S. aureus* biofilm formation. Control, untreated skin PBS only; +*MRSA252*, wounds inoculated with *S. aureus* MRSA252-Rif^R^ only; +*MRSA252*+*phage*, wounds inoculated with *S. aureus* MRSA252-Rif^R^ and subject to phage treatment 4 h post inoculation. **(B,C)** The intensity of fluorescence signal from DAPI or WGA-488 staining measured during CLSM imaging of skin sections. Charts show mean values from three randomly selected regions of skin, in each of three independent replicates. Error bars show standard error of the mean. Statistically significant differences in fluorescence intensity are given by: ^****^P < 0.0001 vs. Control; ^*####*^*P* < 0.0001 vs. MRSA252 only. All images and measurements were taken after 24 h incubation.

To confirm *S. aureus* MRSA252-Rif^R^ forms biofilms in our *ex-vivo* wound model, and understand if phage treatment influenced the formation of these communities, we directly visualized MRSA252-Rif^R^ infected wounds using Confocal Laser Scanning Microscopy. To specifically assess biofilm formation, wounds were stained with the general DNA stain DAPI, and the fluorescent-lectin conjugate WGA-488, which binds to poly-*N*-acetylglucosamine residues present in the exopolymeric matrix of *Staphyloccocus* sp. biofilms. Imaging of wounds 24 h after MRSA252-Rif^R^ inoculation clearly showed biofilms formed within wounds, compared to un-infected controls (Figure 7). Moreover, a clear reduction in the level of MRSA252-Rif^R^ biofilm was evident in wounds treated with phage 4 h post inoculation, and also imaged after 24 h incubation (Figure 7). To support the subjective assessment of confocal images, the intensity of fluorescent signals specific to DAPI staining or WGA-488 biofilm staining, were also measured. This demonstrated a statistically significant reduction in both DAPI and the biofilm-related WGA-488 fluorescent signal in phage treated wounds, compared to infected but untreated wounds (Figure 7).

### Evaluation of accessory gene regulator (*agr*) activity and activation of infection-responsive wound dressing

**Figure 8:**
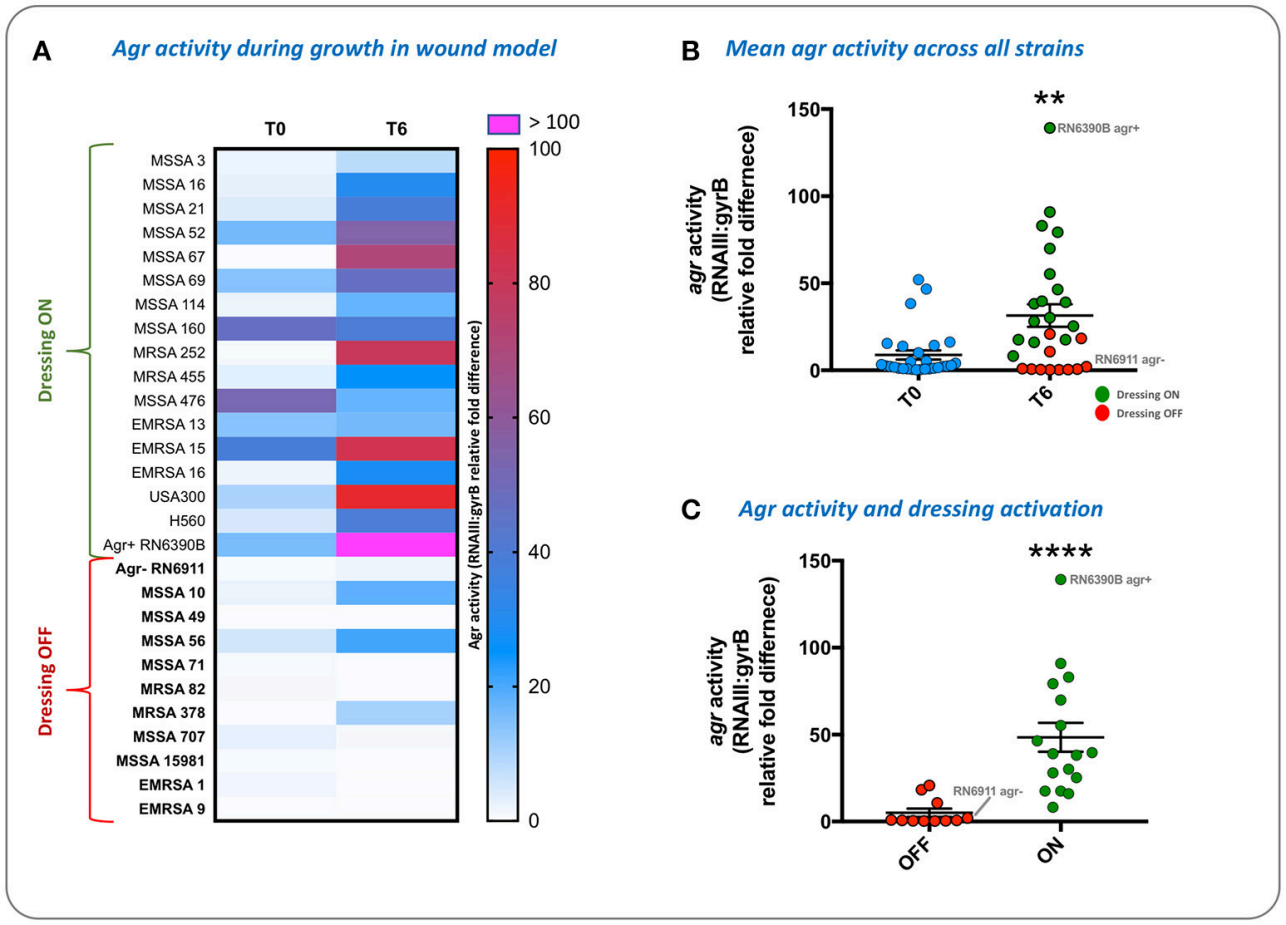
Evaluation of *agr* activity and activation of infection-responsive dressing materials. To understand how *S. aureus* virulence gene regulation was influenced by growth in the porcine wound model, and relate this to activation of prototype infection responsive dressings, the activity of the Accessory Gene Regulator (*agr)* was measured in 28 strains (Table 1). *Agr* activity was assessed by qRT-PCR of *RNAIII* relative to the housekeeping gene *gyrB*. **(A)** Heatmap showing *RNAIII:gyrB* relative gene expression in each strain between time points assessed: Initial inoculum (T0) and after 6 h in growth in wound models (T6). Shading of cells indicates fold-difference in *RNAIII*:*gyrB* expression as indicated by the associated scale. Strains in bold denote those not activating the prototype dressing during the assay. **(B)** Shows mean *RNAIII*:*gyrB* relative expression in all strains, in initial inoculum (T0) and after 6 h growth in wound models (T6). **(C)** Shows mean *RNAIII*:*gyrB* relative expression after 6 h growth in models for strains activating prototype infection-responsive dressings (ON), compared to strains that did not elicit a color change in dressings (OFF). Examples of activated and non-activated dressings are provided in Figure 3. All values show the means of three replicate experiments. Error bars show standard error of the mean. ^**^P ≤ 0.02, ^****^P < 0.0001.

To evaluate the population density-dependant regulation of virulence gene expression during *S. aureus* growth in wound models, and demonstrate the compatibility of our model with molecular genetic assays, we used qRT-PCR to measure the activity of the accessory gene regulator (*agr*) in 28 strains of *S. aureus*. The *agr* system coordinates the population density-dependant expression of a range of virulence genes including toxins (Recsei et al., 1986; Novick, 2003; Laabei et al., 2014). In parallel we tested the ability of each strain to activate prototype infection-responsive dressings, which contain carboxyfluorescein loaded lipid vesicles that are lysed though the action of *S. aureus* toxins regulated by the *agr* (Laabei et al., 2014; Thet et al., 2015; Figure 3). Vesicle lysis and dye release leads to a color change visible to the naked eye that can be used to signal clinically relevant infection (Thet et al., 2015). Evaluation of *agr* activity in cultures used to inoculate wounds at the beginning of experiments (T0) and also after 6 h growth in wound models (T6), revealed much strain-to-strain variation in *agr* activity with some strains exhibiting a reduction in *agr* activity during growth in models (Figure 8A). However, for most strains *agr* activity increased during growth of bacteria in wound models (Figure 8A), and across all strains mean *agr* activity was significantly greater at the end of experiments (Figure 8B). Strains capable of activating prototype dressings also showed a significantly greater mean level of *agr* activity at the end of experiments (T6), compared to strains that did not activate dressings (Figures 8A,C).

## Discussion

The study of burn wound infection and biofilm formation has benefitted immensely from the development and application of a wide range of both *in-vivo* and *in-vitro* model systems (Sun et al., 2008; Dai et al., 2011; Guggenheim et al., 2011; Lebeaux et al., 2013; Roy et al., 2014; Ganesh et al., 2015; Seaton et al., 2015). Collectively the application of these models has considerably advanced our understanding of pathogenesis and biofilm formation in wound infection, but each model has particular merits and limitations, and choice of model is typically a compromise between affordability, ethical considerations, and the aims of a study. There remains considerable scope to improve the range of available models to better facilitate different aspects of wound biofilm study. This includes the development of additional *in-vitro* or *ex-vivo* models with a greater capacity to support the progression of work from early stage discovery of new antimicrobial agents or fundamental processes in biofilm formation, to more complex investigations of host-microbe interactions and wound healing using *in-vivo* models.

Here, we describe the operation of an *ex-vivo* porcine skin model designed to support laboratory study of biofilm formation in burn wounds, and facilitate high throughput screening applications. The core of this model is the burn wound array device (BWAD), used in conjunction with *ex-vivo* porcine skin sections as a substrate for bacterial growth and biofilm formation. The BWAD was shown to generate thermal damage to skin sections similar to partial thickness burns, and both histological analyses and ESEM imaging confirmed damage to the epidermis and underlying collagen consistent with such injuries. The BWAD also ensures consistency between wounds within the array, and those generated on different skin sections, by allowing factors such as temperature, pressure, and time used to generate thermal damage to be easily controlled and standardized during model operation. There is also considerable scope to vary these parameters to tailor models according to individual requirements. The wounds generated were also shown to be capable of supporting bacterial growth and biofilm formation without addition of further exogenous media, and the model is compatible with a range of common analytical techniques for monitoring bacterial growth, metabolic activity, biofilm formation, and gene expression. These included specific staining and visualization of biofilms using confocal laser scanning microscopy, and excellent reproducibility was observed between biological replicates in all assays employed in this study.

Collectively these features provide a high-throughput model with good reproducibility for the study of burn wound infection and biofilm formation, which should also provide an improved representation of the wound environment, compared to models using abiotic surfaces or mammalian cell monolayers. It is also notable how other *in-vitro* wound models have been used in conjunction with *in-vivo* studies to improve performance and study design. In particular, several studies have described the use of *in-vitro* systems to prepare biofilms for transplantation into *in-vivo* wound models (Sun et al., 2008; Dalton et al., 2011; Guggenheim et al., 2011). In this context it seems logical that biofilms preformed on *ex-vivo* skin sections, rather than plastic substrates, could be more compatible with *in-vivo* applications.

Nevertheless, as an *ex-vivo* model, notable limitations will be inherent in the system we describe here, restricting types of study for which this is a suitable model (Sun et al., 2008; Guggenheim et al., 2011; Lebeaux et al., 2013; Ganesh et al., 2015). In the case of the preliminary proof-of-concept studies we present in this manuscript, only single species biofilms of *S. aureus* were evaluated (discussed in detail below). However, this aspect of the work described does not necessarily reflect a deficiency in the model *per se*, and previous studies using our more basic porcine skin model showed that growth of numerous clinically relevant species are supported (Thet et al., 2015), highlighting the capacity to develop protocols for growth of polymicrobial biofilms in this model.

In contrast, a lack of host response and the relatively short duration (up to 4 days) over which experiments with our *ex-vivo* model may be conducted, are clearly key limitations also found in *in-vitro* models (Lebeaux et al., 2013; Roy et al., 2014; Ganesh et al., 2015). These features significantly reduce the utility of our *ex-vivo* system to study fundamental aspects of the interplay between biofilm formation and wound healing, in terms of host response and wound repair processes, as well as chronic long-term wound infections (Roy et al., 2014; Ganesh et al., 2015). As an example, Roy et al. (2014) have recently described the use of an *in-vivo* porcine model of chronic burn wound infection to study host response to biofilm formation (Roy et al., 2014). Using this model the presence of biofilm-induced microRNAs in skin at the wound periphery were identified, which silenced genes involved in tight junction formation, and presumably influence wound closure (Roy et al., 2014). In this case, the use of an *in-vivo* porcine model is clearly required to model long-term chronic infections with biofilm formation over a period of weeks, and also evaluate impact on host wound healing processes including immune response and gene expression.

However, while *in-vivo* studies like those described by Roy et al. (2014) are clearly indispensable for fundamental investigations of wound healing and host response to infection, they are less well-suited to the early stage discovery and evaluation of novel anti-microbial agents for control of wound infection. Conversely, because our *ex-vivo* model is relatively high throughput, low cost, and does not use live animals, it is well-suited to the early stage study and evaluation of approaches to control bacterial growth or biofilm formation, before more complex and ethically contentious experiments are considered (Sun et al., 2008; Guggenheim et al., 2011; Lebeaux et al., 2013; Ganesh et al., 2015). This not only includes screening of compound libraries, and the evaluation of susceptibility across many isolates (in terms of minimum bactericidal concentration or minimum biofilm eradicating concentration), but also the initial evaluation of novel and untested approaches to combat biofilm formation. The use of animal models for these initial studies is difficult to justify from both ethical and financial perspectives, without reference to initial data supporting the potential to control biofilm formation or microbial growth. Moreover, skin sections used in our model may be derived from considerably fewer animals than typically required for *in-vivo* experiments, and from those killed for other purposes (as was the case for this work), including reasons unrelated to scientific research.

To demonstrate the potential utility of this model for early stage development of novel infection control strategies, we evaluated the ability of bacteriophage (or phage) to inhibit biofilm formation by *S. aureus*. Phage are bacterial viruses that specifically infect and kill bacteria, and represent a promising alternative to antibiotics, particularly for infections involving bacterial biofilms (Curtin and Donlan, 2006; Lu and Collins, 2007; Carson et al., 2010; Fu et al., 2010; Alves et al., 2014, 2016; Lehman and Donlan, 2015; Nzakizwanayo et al., 2016). Of particular relevance in this context are strategies phage have evolved in order to disrupt bacterial biofilms to access bacterial hosts growing in these communities (Hughes et al., 1998; Sutherland et al., 2004; Donlan, 2009; Harper et al., 2014), and a number of recent studies have begun to highlight the potential for phage to be used to control wound infection (Busch et al., 2000; Marza et al., 2006; McVay et al., 2007; Kumari et al., 2009a,b; Rose et al., 2014).

Data generated in this study using our *ex-vivo* model showed that a single phage treatment was able to reduce *S. aureus* biofilm formation when assessed at the 24 h timepoint. More detailed examination by selective staining and confocal microscopy supported the formation of biofilms in wounds, and a significant reduction in biofilm formation at the 24 h time point after a single dose of phage. However, it was notable that in models run for 48 h and subject to successive doses of phage, viable cell counts were not significantly reduced compared to untreated controls. This was despite a significant reduction in metabolic activity in XTT assays, and clear evidence that phage continued to proliferate in wounds over this period. This discrepancy may relate to the different ways in which each method (viable cell counts and the XTT assay) assesses the bacterial population. It is likely that the reduced metabolic activity observed at 48 h is not fully related to reduced numbers of viable cells, but an indication of prevailing environmental parameters in these longer running experiments. In particular, cells located deeper within mature and more established biofilms are known to enter a quiescent state with low metabolic activity, distinct from planktonic cells or those at earlier stages of biofilm formation, or at the biofilm periphery (Donlan and Costerton, 2002; Zhao et al., 2013). There is also potential for development and selection of phage resistant cells over this time period, which could contribute the differences in viable cell counts and XTT assays at these later time points.

Although phage are clearly still able to proliferate over the course of longer 48 h experiments, collectively our data suggests that this replication, and additional phage doses, is not sufficient to further reduce the bacterial population. This likely indicates that host-phage interactions may have reached an equilibrium, and may also reflect a reduced susceptibility of more mature biofilms to phage infection. Collectively, these data may indicate that phage are more effective if applied early in infection or prophylactically, in order to inhibit biofilm formation, but less effective if biofilms have already developed in the wound. Similar findings have also been noted for studies of phage therapy in other biofilm related infections (Nzakizwanayo et al., 2016). Alternatively, it should be noted that although the combined use of multiple phage as a cocktail would seem a logical approach to enhance activity and offset the development of resistance, in reality there remains the potential for antagonistic interactions between individual phage which could reduce efficacy of the cocktail in controlling the bacterial population. Nevertheless, these initial investigations provide good preliminary insights in the use of phage to control biofilms in this setting, and demonstrate how this *ex-vivo* system may be used in early stage investigations to select promising phage, or phage combinations for further evaluation in more complex *in-vivo* models.

Our model also provides a useful and accessible platform for studies seeking to understand basic features and molecular genetic aspects of biofilm formation, with the view to identifying new targets for control or detection of biofilm formation, or to better understand this process in bacteria. This could include approaches such as the large-scale screening of mutant libraries to identify those defective in traits of interest (where use of animals is generally not feasible or justifiable); or the more detailed phenotypic characterization of mutants recovered from competitive *in-vivo* screening of mutant pools representing genome wide saturating mutagenesis of target pathogens, such as Tn-seq based investigations (Lebeaux et al., 2013; Zhao et al., 2013). Here, we used our model to monitor bacterial gene expression during biofilm formation, demonstrating its compatibility with challenging molecular biology techniques such as recovery of RNA.

This work showed that in *S. aureus* population density-dependant expression of virulence attributes is evident in our model, as indicated by *agr* activity, and activation of prototype infection-responsive dressings which rely on *agr* mediated toxin production (Laabei et al., 2014; Thet et al., 2015). The results obtained in this study are congruent with our previous studies of vesicle lysis by *S. aureus* with regard to toxin production and *agr* activity. For the most part, strains we evaluated in earlier studies, and also in the current work, produced comparable results. Strains RN6911, MRSA71, MRSA378, and MRSA707 failed to activate dressings while MRSA69, MRSA252, and RN6390B elicited a clear color change both in our models and previous studies of vesicle lysis in broth cultures (Laabei et al., 2014). In addition, the overall differences in *agr* activity in these strains was also in line with our previous work (Laabei et al., 2014), in that strains failing to activate dressings or lyse vesicles exhibited little or no notable *agr* activity. This general pattern was also evident in the wider panel of strains tested in this study with respect to dressing activation.

Notable exceptions, however, were strains MRSA10, MRSA56, and MRSA378, which exhibited an increase in *agr* activity during growth in wound models comparable to the majority of other strains capable of activating dressings, but failed to activate dressings during the 6 h test period. For MRSA378 the lack of dressing activation and the general lack of *agr* activity measured in broth cultures at the start of experiments was in keeping with observations we reported previously in broth cultures (Laabei et al., 2014). This was contrasted by the increased *agr* activation in this strain at the end of the wound model experiments, which was not consistent with our previous data. These observations suggest that features of the wound model not replicated in abiotic assays (such as broth cultures) support increased *agr* activity in some strains. Discrepancies between *agr* activity and dressing activation in these strains may therefore be the result of mutations in other genes, aspects of toxin translation and export, or arise from differences in the rate or specific timing at which *agr* expression increases and the total level of toxins generated during these assays. This may also include the relative fitness of different strains and in the wound model and ability to gown to levels sufficient to trigger *agr* activation, which should be explored in further studies.

Collectively these observations suggest our wound model can provide insights into the regulation of processes such as virulence factor production and biofilm formation not obtained in *in-vitro* models. This is also supported by previous studies in our initial low-throughput *ex-vivo* model, which indicated that for some species, clinically relevant phenotypes not observed in *in-vitro* systems were apparent in the *ex-vivo* model (Thet et al., 2015). This likely stems from the use of porcine skin tissue as a primary substrate for growth and biofilm formation, which is a major difference to other popular *in-vitro* wound models which utilize abiotic substrates (Sun et al., 2008; Guggenheim et al., 2011), and further highlights the benefits of using an *ex-vivo* system for this type of study. This should also provide more robust data on which to select agents for further study, inform the design of subsequent *in-vivo* studies, and better support basic molecular genetic studies of the infection process, which will in turn contribute to the more effective use of animals in research and the reduction of numbers used.

## Ethics statement

This study utilized only surplus tissues from animals culled for other scientific research. The use of surplus porcine skin was considered and supported by the Pirbright Institute's Animal Welfare and Ethical Review Board, and was compliant with the UK Animals Scientific Procedures Act 1986 (ASPA).

## Author contributions

DA, SB, BJ, and AM conceived the study. DA, SB, JN, PasS, PaoS, CD, RW conducted the experiments. All authors contributed to design and conduct of the study, and analysis and interpretation of the data. DA, BJ, SB, and JN wrote the manuscript and all authors edited the manuscript.

### Conflict of interest statement

The authors declare that the research was conducted in the absence of any commercial or financial relationships that could be construed as a potential conflict of interest.
